# Facing Trauma and Surgical Emergency in Space: Hemorrhagic Shock

**DOI:** 10.3389/fbioe.2022.780553

**Published:** 2022-07-01

**Authors:** D. Pantalone, O. Chiara, S. Henry, S. Cimbanassi, S. Gupta, T. Scalea

**Affiliations:** ^1^ Department of Experimental and Clinical Medicine, Fellow of the American College of Surgeons, Core Board and Head for Studies on Traumatic Events and Surgery in the European Space Agency-Topical Team on “Tissue Healing in Space Techniques for Promoting and Monitoring Tissue Repair and Regeneration” for Life Science Activities Agency, Assistant Professor in General Surgery, Specialist in Vascular Surgery, Emergency Surgery Unit–Trauma Team, Emergency Department–Careggi University Hospital, University of Florence, Florence, Italy; ^2^ Fellow of the American College of Surgeons, Director of General Surgery–Trauma Team, ASST GOM Grande Ospedale Metropolitano Niguarda, Professor of Surgery, University of Milan, Milan, Italy; ^3^ Fellow of the American College of Surgeons, Director Division of Wound Healing and Metabolism, R Adams Cowley Shock Trauma Center University of Maryland, Baltimore, MD, United States; ^4^ Fellow of the American College of Surgeons, EMDM, Vice Director of General Surgery-Trauma Team, ASST GOM Grande Ospedale Metropolitano Niguarda, Milan, Italy; ^5^ Fellow of the American College of Surgeons, R Adams Cowl y Shock Trauma Center, University of Maryland, Baltimore, MD, United States; ^6^ Fellow of the American College of Surgeons, The Honorable Francis X. Kelly Distinguished Professor of Trauma Surgery.Physician-in-Chief, R Adams Cowley Shock Trauma Center, System Chief for Critical Care Services, University of Maryland Medical System, University of Maryland, Baltimore, MD, United States

**Keywords:** hemorrhage, trauma, hemostats, blood substitutes, space missions

## Abstract

Although the risk of trauma in space is low, unpredictable events can occur that may require surgical treatment. Hemorrhage can be a life-threatening condition while traveling to another planet and after landing on it. These exploration missions call for a different approach than rapid return to Earth, which is the policy currently adopted on the International Space Station (ISS) in low Earth orbit (LEO). Consequences are difficult to predict, given the still scarce knowledge of human physiology in such environments. Blood loss in space can deplete the affected astronaut’s physiological reserves and all stored crew supplies. In this review, we will describe different aspects of hemorrhage in space, and by comparison with terrestrial conditions, the possible solutions to be adopted, and the current state of the art.

## Introduction

Treatment of unforeseen health events in space is a major concern that can jeopardize crew health and mission success (Hamilton et al., 2008; Alexander 2016; Patel et al., 2020). This concern has given rise to several studies on open surgery, laparoscopic surgery, and robot-assisted surgery as well as studies of human physiology in microgravity (for a review of the literature, see Panesar et al., 2018 (Melton et al., 2001; Campbell 2002; Dawson, 2008; Kirkpatrick et al., 2009a; Kirkpatrick et al., 2009b; Kirkpatrick et al., 2009c; Doarn et al., 2009; Pletser et al., 2009; Haidegger et al., 2011; Canga et al., 2016; Ashrafian et al., 2017; Panesar and Ashkan 2018; Baker et al., 2019; Hinkelbein et al., 2020; Kirkpatrick et al., 2020; Robertson et al., 2020). Although in space the risk of trauma is low, objects in movement conserve their mass and still carry a kinetic energy (Komorowski et al., 2018; STEMonstrations 2022) so that damages to the human body are likely to occur. It must be remembered that kinetic energy greatly increases due to velocity rather than mass, consequently a small increase in speed will result in an increased risk of injury (Komorowski et al., 2018; STEMonstrations 2022). Although so far the likelihood of severe trauma or surgical emergency has been considered low, crushing trauma and penetrating trauma as well as unpredictable events requiring surgical treatment are considered a major concern (Hamilton et al., 2008). Their impact affects an organism that has to find a new balance for the absence of gravity and hence is more fragile even in case of minor trauma than on Earth. Crushing trauma and penetrating trauma can directly harm the astronaut and also damage the protective suit, a potentially catastrophic event as it can cause spacesuit decompression or even ignition (Khorasani-Zavareh et al., 2014; Panesar and Ashkan 2018). Even if to date there have been no accounts of major hemorrhage in space, it can be a life threatening condition during a long term mission or mission to Mars. These exploration missions required a different policy than rapid return to Earth, currently adopted on the International Space Station (ISS) in low Earth orbit (LEO). Although the likelihood of hemorrhage may be prevented through vehicle design adaptation, adequate crew training, and accurate medical crew selection, the occurrence of significant blood loss must be taken into account. Launching, landing and docking or extra vehicular activity (EVA) are dynamic stages at risk of truama. A severe bleeding after landing on another celestial body would have consequences that are difficult to predict given the still poor knowledge of human physiology in such environments, (Roth 1968). On Earth, trauma severity, either blunt or penetrating, can be generally defined by a mechanical damage to the body caused by an external force (Khorasani-Zavareh et al., 2014).

Kirkpatrick had already reported in 2005 (Kirkpatrick et al., 2005) that hemorrhage is the leading cause of potentially preventable deaths on Earth and, in space exploration, considered to be the most significant risk for astronauts, followed by infections (Kirkpatrick et al., 2009c). Blood loss in space can deplete the physiological reserves of the affected astronaut for lack of the standard requirements normally available on earth. In addition, such an occurrence is not only a challenging situation for any individual astronaut, but it can deplete crew resources (Hamilton et al., 2008) as well, as we will explain in this review.

## Materials and Methods

A literature review was done to identify publications on PubMed and Medline, Embase. The following key words were searched: “hemorragic shock,” “microgravity,” “zero gravity,” “astronauts,” “blood,” “transfusion,” “hemorrhage,” “space missions,” and “mission to Mars.” Collected papers were selected according to the following criteria: English language or, if in another language, be provided from online translation tools or at least come in the form of a structured English abstract. References obtained were crosschecked for additional relevant publications.

## In Missions

### The Space Environment

Presently, in spaceflight, the most critical moments are launch and landing due to the stressful conditions crew members are under in the spacecraft. In fact, human losses in space missions have occurred in those phases (Barratt 2019). Inside the spacecraft, the Environmental Control and Life Support System (ECLSS) maintains the atmosphere steady, normoxic, and normobaric while at neutral temperatures. However, the carbon dioxide concentration inside the cabin is on average 10 times higher than on the ground (0.3%–0.5%). The environment outside the spacecraft is the most hostile ever experienced before, nil barometric pressure, high levels of radiation, and extreme temperatures (−150°C to +120°C) (Komorowski et al., 2016; Barratt 2019). An immediate hazard is caused by a loss of cabin pressure (e.g., in case meteorite or satellite debris were to hit the station), fire, release of toxic substances, or malfunction of the ECLSS (Environmental Control and Life support System) (Kirkpatrick et al., 2005; Kirkpatrick et al., 2009c). In space, any of these events could potentially cause and aggravate a potential injury.

### Hints on Changes in Human Body Physiology in Space Related to Hemorrhagic Shock

In space, numerous changes occur in human body physiology (Table 1). Some of these changes may particularly affect the body’s response to bleeding, mainly the cardiovascular system. The cardiovascular system is involved in response to a hemorrhagic event. The redistribution of body fluids toward the head is a well-known phenomenon in microgravity (Komorowski et al., 2016; Barratt 2019; Nowak et al., 2019). The fluid shift initiates at the launch position with the lower limbs raised above the thoracoabdominal coronal plane, a condition that continues during orbit, producing a displacement of blood and other fluids from the lower limbs to the torso and head (Williams et al., 2009; Nowak et al., 2019). When astronauts arrive in space, the gravitational pull that drives the circulation toward the feet stops working and fluids shift toward the head and torso (Alexander 2016). This shift and the compensation by the cardiovascular system can make the human body more subject to potentially harmful cardiovascular effects. The compensation for volume redistribution includes the activation of central baroreceptors (Convertino 2003; Di Rienzo et al., 2008; Panesar and Ashkan 2018) and suppression of the renin–angiotensin–aldosterone axis with the release of the atrial natriuretic peptide. Salt and water are excreted, with a reduction in plasma volume and a transient increase in hematocrit levels. A decrease in both erythropoietin secretion and red cell mass is also present, with a reduction in blood volume (Panesar and Ashkan 2018) (see Panesar and Ashkan (2018)) for the review of the literature). The decrease in cardiac workload in prolonged spaceflight may reduce the overall myocardial mass (Convertino 2009; Demontis et al., 2017; Panesar and Ashkan 2018), but despite the loss of contractile mass, ejection fraction and arterial pulse wave velocity are preserved. The human body is able to compensate the fluid shift through diuresis, with a reduction of extracellular fluid and plasma volume, an event that produces a decrease in body mass during the first 30 days (Panesar and Ashkan 2018).

**TABLE 1:** Effects of microgravity that could affect physiologic response to hemorrhage.

Fluid redistribution	Fluid redistribution is followed by decrease in blood volume, cardiac size, and aerobic capacity with a post-flight orthostatic intolerance known as “cardiovascular deconditioning.” The redistribution is caused by fluid shifts from the intravascular to the interstitial spaces due to lower transmural pressure for reduced compression of all tissues by gravitational forces, by fluid shifts from intravascular to muscle interstitial spaces due to lower muscular tone required to maintain the body posture. Decreased diuresis in the initial phases of space flight is due to the increased retention after stress-mediated sympathetic activation Antonutto and di Prampero (2003), Demontis et al. (2017), Tanaka et al. (2017), Iwase et al. (2020), Gallo et al. (2020)
Blood	-Reduction in circulating blood volume (a loss of 10–23% of circulating blood volume) resulting in an earth hypovolemic state Kirkpatrick et al. (2001), Kirkpatrick et al. (2005), Diedrich et al. (2007), Nowak et al. (2019)
	-Reduction in red cell mass (10–20% with respect to the preflight baseline) although this effect diminishes with the increase in mission duration Nowak et al. (2019)
	-Missions that last more than 6 months cause an increase in red blood cells, platelets, and hemoglobin concentration, probably related to reduction in plasma volume Nowak et al. (2019), consequent blunting of the baroreceptor response, and vasodilatation with a decrease in heart rate and blood pressure
	-The cephalad fluid shift causes an increase in venous return with increased stroke volume that produces alterations in the autonomic and endocrine systems designed to control the cardiovascular functions Iwase et al. (2020)
Heart	-Cardiac atrophy and reduced cardiac output Nowak et al. (2019), Demontis et al. (2017), Iwase et al. (2020)
Neuroumoral and cardiovascular system	-Reset of the working parameters of the neuroumoral and cardiovascular system
	-Possible global resetting of the centronomic nervous system with either a beta receptor bias or impaired receptor sensitivity resulting in an overall attenuation of the cardiac chrono tropic response Baker et al. (2019)
	-Attenuation of the aortic cardiopulmonary and carotid baroreflex responses to hypotension would presumably decrease the ability to respond appropriately to hypovolemic stress Baker et al. (2019).
Vessels	Muscle sympathetic nerve activity (MSNA) is designed to control the vasomotor function of the muscular bed Iwase et al. (2020), and its response to blood pressure changes against gravitational stress. MSNA responds also to the loading or unloading of the cardiopulmonary receptors when stimulated by the cephalad fluid shift Iwase et al. (2020).

### Cardiac Function

Cardiac function adapts to the fluid shift and to the alterations by increasing cardiac output (Demontis et al., 2017; Panesar and Ashkan 2018). In spaceflight, the cardiovascular system is affected by one of the major alterations in human physiology. Over time, the shift of fluids from the lower body to head and torso produces loss of ventricular mass (cardiac atrophy) (Perhonen et al., 2001; Demontis et al., 2017; Evans et al., 2018), decreased sensitivity of the carotid-cardiac (vagal) baroreflex (Williams et al., 2009; Norsk 2014), and a greater responsiveness of sympathetic neural activity to inflight simulations of standing (Williams et al., 2009; Demontis et al., 2017). The effect is a decreased blood pressure and elevation of cardiac output throughout flight (Perhonen et al., 2001; Ertl et al., 2002a; Ertl et al., 2002b; Norsk 2014; Evans et al., 2018).

Vasodilation, present in space permanence, may reduce (Norsk et al., 2015; Norsk 2020) plasma volume and associated cardiovascular effects. This scenario is called cardiac deconditioning (Demontis et al., 2017), and decreased compensatory responses, exacerbated by relative hypovolemia and anemia manifesting during spaceflights (Nowak et al., 2019). However, increased levels of red blood cell platelets and higher hemoglobin concentration are reported in long duration flights, but these effects are probably linked to the plasma volume decrease occurring in space (Smith 2002; Kunz et al., 2017). The altered physiologic conditions of the cardiovascular system may result in a decreased ability to respond to blood loss in weightlessness. Hence, in case of hemorrhage, the time to intervene effectively is probably shorter (Kirkpatrick et al., 2009b; Williams et al., 2009) and rescue must be rapid, making fluid resuscitation a priority.

### Autonomic Nervous System and Hypothalamic–Pituitary–Adrenal System in Space Conditions—Hints on Their Possible Role in Hemorrhage

The hypothalamic–pituitary–adrenal HPA (axis) plays an important role in the adaptation to stress (Buckey and Homick 2002; Smith 2006; Welt et al., 2021). It is the most important interconnection between the nervous system and the endocrine system. The activation of the HPA axis leads to the secretion of glucocorticoids, which act on multiple systems and organs to redirect the energy resources necessary to meet a real need or even a possible need that could occur. The HPA axis response to stress is driven primarily by neural mechanisms, with responses that may be inhibited by feedback (e.g., production of glucocorticoid hormones). In a stressful situation, the axis mediates the effect of stress factors by regulating numerous physiological processes, such as metabolism, immune responses, and activation of the autonomous nervous system (ANS) (Chouker 2020; Tobaldini et al., 2020). In the presence of hypovolemic shock, there is an activation and release of adrenaline and noradrenaline by the adrenal medulla and glucocorticoid hormones by the adrenal cortex, in addition to glucagon from the pancreas (Smith 2006; Mandsanger et al., 2015; Herman et al., 2016; Crucian et al., 2018; Chouker 2020; Tobaldini et al., 2020; Kageyama et al., 2021).

ANS regulates the cardiovascular system and controls visceral functions in order to maintain homeostasis and the homeodynamic state of the body. It is also an interface between the body, the central nervous system (CNS), and external stimuli (Mandsanger et al., 2015; Chouker 2020; Tobaldini et al., 2020). Its sympathetic branch plays a role in the control of many activities, for example, cardiovascular, gastrointestinal, pulmonary, cutaneous, genitourinary, and immune. Despite being defined as an “autonomic” system, there are complex control mechanisms that act both centrally and peripherally (Mandsanger et al., 2015; Herman et al., 2016; Chouker 2020; Tobaldini et al., 2020) in pathological conditions such as hypertension, heart failure, and myocardial infarction stress (Malliani 2000; Wallin and Charkoudian 2007; Herman et al., 2016; Tobaldini et al., 2020). ANS plays an important role in the regulation of the vegetative state and also in the modulation of the responses of the immune system (Kirkpatrick et al., 2009b; Mandsanger et al., 2015; Chouker 2020; Tobaldini et al., 2020), metabolism, and inflammation (Kirkpatrick et al., 2009b; Chouker 2020; Tobaldini et al., 2020; Welt et al., 2021), suggesting an integration at different levels of control (Kirkpatrick et al., 2009b; Mandsanger et al., 2015; Chouker, 2020; Tobaldini et al., 2020).

When we talk about modifications present in space, we refer mainly to the studies conducted on parabolic flights. In them, there are short phases in which there is a change in severity. In this way, the effects of the different phases of flight on hemodynamics and the cardiovascular system were studied: 1) 1 g (before and after each parabola), 2) hypergravity during the ascending part, 3) microgravity phase at the apex of parabola, 4) hypergravity during the descending part of parabola, and 5) 1 g at the end of the parabola (Iwase et al., 2020).

In fact, these experiments simulate the hypergravity and microgravity characteristics of space missions (Criscuolo et al., 2020). These studies were born with the intent to explore the effects of different gravity levels (zero, lunar, and Martian gravity) on cardiovascular and autonomous control for missions to Mars in the near future. During the parabolic flight, the correlations between the level of gravity and the cardiovascular autonomic modulation have been at the center of many studies (Widjaja et al., 2015). Despite this, to the best of the authors’ knowledge, these effects have not yet been sufficiently studied to predict what might happen in the event of a severe hemorrhagic shock.

## Cardiovascular Autonomic Control During Space Flights

The cardiovascular function is profoundly influenced by microgravity and thus also by autonomous cardiovascular control. In the case of space flights, each component of this system can be affected by the new conditions to which it is subjected. It is known that in microgravity there is a reduction in cardiac mass and vascular function is worsened by presenting stiffened arteries and affected by endothelial dysfunctions (Kirkpatrick et al., 2009b; Widjaja et al., 2015; Alexander 2016; Hughson et al., 2018; Criscuolo et al., 2020; Iwase et al., 2020). In other words, the cardiovascular system shows a reduced ability to respond to stressful situations. Consequently, it is presumable that in the event of a hemorrhagic shock, the response capacity is compromised in space. There is currently no evidence on this topic, but it is presumable that all the aforementioned modifications would force the cardiovascular system to a non-optimal performance. Furthermore, on Earth, there are neither cosmic radiations nor microgravity, which instead act synergistically in space (Jones et al., 2019). It is important, in fact, that adequate shielding for deep space flights is implemented because even low doses of radiation are able to increase the risk of cardiovascular mortality (Jones et al., 2019).

### Hints on Inotropes and Other Medications for Space Missions

On the ground, the main indication in massive bleeding shock is surgery, i.e., damage control surgery (Ball 2017), which must be applied as soon as possible (Ertl et al., 2002a; Ertl et al., 2002b; Kirkpatrick et al., 2005; Wallin and Charkoudian 2007). Persistent hypotension after fluids administration (ATLS protocol) (American Committee for Trauma-American College of Surgeons 2018) is treated with vasopressors (epinephrine and norepinephrine) to improve systolic pressure. However, drugs (Standl et al., 2018) with different targets are available in case of failure of inotropic infusion. For example, dobutamine can be used in cardiogenic shock and in any type of shock with insufficient ventricular pump function. Other drugs (Standl et al., 2018) such as milrinone, levosimedan, vasopressin, glyceryl trinitrate, and sodium nitroprusside have found applications in different types of shock, mainly cardiogenic, with the exception of cafedrine hydrochloride and theoadrenaline hydrochloride that are used for neurogenic shock. However, a specific review for this topic should be the best option to discuss it in depth.

### Hemorrhage Control in Space

The ability to control hemorrhage after a traumatic injury in space is crucial in astronaut’s health care (Table 2). The crew must continue its mission autonomously, and any medical care is performed in a setting where resupply, evacuation, and communication are difficult (Hamilton et al., 2008). In space, medical systems as well as supplies, equipment, and crew training are limited.

**TABLE 2:** Approach to hemorrhage control in space and on Earth. Research studies conducted on the ISS (Neurolab Missions).

	Space	Earth
Initial treatment	Applications of ATLS American Committee for Trauma-American College of Surgeons (2018) protocols for airway protection, drainage of hemopneumothoraces, and initial resuscitation. Needs to rapidly localize and address major hemorrhage Kirkpatrick et al (2009c)	Applications of ATLS American Committee for Trauma-American College of Surgeons (2018) protocols for airway protection, drainage of hemopneumothoraces, and initial resuscitation. Needs to rapidly localize and address major hemorrhage
Diagnostics	Ultrasounds (FAST) Kirkpatrick et al. (2007) (possible detection of an alarming rate of bleeding (on earth bleeding rates over 25 ml/min: estimated window before death −2 h)	Ultrasounds (FAST) Kirkpatrick et al. (2009c), Kirkpatrick et al. (2007)
	In space, intracavitary, thoracic, and abdominal hemorrhage will be more difficult to detect than on the earth and will require higher levels of skills and resources for treatment Kirkpatrick (2017a), Kirkpatrick (2017b)	CT scan (hemodynamically stable patient) ATLS American Committee for Trauma-American College of Surgeons (2018)
Blood administration	Lyophilized blood products, hemoglobin-based oxygen carriers (HBOCs) Nowak et al. (2019). The use of human blood is to be excluded for now	Early use of fresh warm whole blood transfusions and of blood products Nowak et al. (2019)
	Astronauts have an estimated 15% decrease in circulating red blood cells and plasma on-orbit, the equivalent of Class I hemorrhage Alexander (2016)	
Cardiac output	(Animal studies): changes in cardiac output and blood pressure when subjected to + G centrifugation (Antonutto and di Prampero, 2003; Komorowski et al., 2016; Demontis et al., 2017; Tanaka et al., 2017; Barratt 2019; Nowak et al., 2019; Iwase et al., 2020; Gallo et al., 2020)	
Intravenous fluid administration	Gravity absence no longer pulls fluids out of fluid bags into the body without an external force (risks for bubble formation). It is difficult to control the rate of fluid administration using such techniques (Antonutto and di Prampero, 2003; Komorowski et al., 2016; Demontis et al., 2017; Tanaka et al., 2017; Barratt 2019; Nowak et al., 2019; Iwase et al., 2020; Gallo et al., 2020)	
Surgery	To date, no information on possible major surgery for severe trauma, use of damage control surgery Jenkins et al. (2014), Lamb et al. (2014), Kirkpatrick et al. (2017a), Kirkpatrick et al. (2017b), and damage control resuscitation	Damage control surgery Jenkins et al. (2014); Lamb et al. (2014), Kirkpatrick et al. (2017a), Kirkpatrick et al. (2017b), damage control resuscitation, staged surgery, and multidisciplinary team activation
		Junctional and compressive device Gordy et al. (2011), Rappold and Bochicchio (2016); Chiara et al. (2018); Huang et al. (2020); Tompeck et al. (2020)
	No information on the use of other techniques as the role of the resuscitative endovascular balloon for occlusion of the aorta (REBOA) Brenner et al. (2018), Cannon et al. (2018) or angioembolization techniques	Balloon occlusive device for the aorta (REBOA) Brenner et al. (2018), Cannon et al. (2018)
		Angioembolization techniques
	Also the use of hemostatics Gordy et al. (2011), Rappold and Bochicchio (2016), Chiara et al. (2018), Huang et al. (2020), Tompeck et al. (2020) possible on Earth, lacks strong evidence relatively to space missions	Hemostats Gordy et al. (2011), Rappold and Bochicchio (2016), Chiara et al., 2018, Huang et al. (2020), Tompeck et al. (2020)
		Intracavitary foam Rago et al. (2016)
		Expandable hemostatic sponge and other devices Huang et al. (2020), Tompeck et al. (2020)

It must be underlined that estimates of traumatic injuries are mainly based on terrestrial populations and do not include spaceflight data. They are extrapolated to the astronauts and referred to the environment of the International Space Station (ISS). In addition, estimates do not account for injury risks due to long duration surface operations under the influence of gravity, and for increased risks of acute radiation sickness (ARS) (Jones et al., 2019), both conditions are expected in Moon or Mars missions (Nowak et al., 2019).

In case of medical issues, stabilization and expeditious evacuation back to Earth are the present policy (Hamilton et al., 2008; Kirkpatrick et al., 2009c; Hodkinson et al., 2017) on the ISS, although standard advanced trauma life-support (ATLS) (American Commettee for Trauma-American College of Surgeons 2018), intravenous insertions for infusion, endotracheal intubation, and chest tube placement are practices that the crew medical officer (CMO) must know (Hamilton et al., 2008; Kirkpatrick et al., 2009c; Hodkinson et al., 2017). A major traumatic hemorrhage in space would be catastrophic. Hemorrhage can occur either externally, from open wounds, or internally, into closed anatomic spaces. It can be categorized as compressible and non-compressible, depending on the location (Kirkpatrick et al., 2005; Ball 2017; American Commettee for trauma-American College of Surgeons 2018). Non-compressible torso hemorrhage (NCTH), coming from the torso vessels, the pulmonary parenchyma, solid abdominal organs, or disruption of the bony pelvis, is occult and not treatable by simple compression and therefore be fatal (Ball 2017). On Earth, a blunt, polytrauma patient can be a challenge due to difficulty in detecting internal bleeding and could require advanced surgical skills and more dedicated devices than with external bleeding (Brenner et al., 2018; Cannon et al., 2018). Vice versa, in weightlessness, this type of injury can be devastating and seemingly impossible to treat, despite the help of ultrasound in diagnosis (Hamilton et al., 2008; Kirkpatrick et al., 2009c; Pletser et al., 2009; Alexander, 2016; Garrigue et al., 2018; Mashburn et al., 2019) It can absolutely drain a mission’s resources with no chance of resupply. The need for blood supplies in resuscitation is another major issue in space. It is known that morbidity and mortality resulting from hemorrhage decrease with the use of blood products and the derivatives of blood (Nowak et al., 2019). Therefore, blood transfusion, already a life-saving procedure on Earth, should be considered all the more important in space.

In an article, Nowak et al. (2019) reported on the need for research on alternative blood products in hostile environments. For example, lyophilized blood components like plasma that undoubtedly have advantages over liquid storage for mass, volume, and limited shelf life (Pusateri et al., 2016; Garrigue et al., 2018; Nowak et al., 2019).

Another possibility is the use of hemoglobin-based oxygen carriers (HBOCs) (Moore et al., 2009; Weiskopf et al., 2017; Nowak et al., 2019), which are artificial red blood cell substitutes able to deliver oxygen and provide volume expansion. They require little preparation; however, mass and volume are similar to red packed cells, occupying room in the space craft. Additionally, they are still experimental (Nowak et al., 2019).

In space, limitations due to mass, volume, and power (Nowak et al., 2019) affect blood storage capability which requires a significant use of refrigeration power. Refrigeration is also associated with a limited shelf life, 35 days at 1–6°C, while a trip to Mars lasts at least 6 months. However, to date, the most practical application for transfusion in space is fresh whole blood despite the limits set by circulatory physiology, resupply issues, and personal restraints in the spacecraft (Nowak et al., 2019). Other issues have to be taken into account in space (Nowak et al., 2019) such as the following:

-The need to restrain CMO and patients during spaceflights.
-The risk of venous thromboembolism after a venous line insertion due to loss of stratification of liquids and gases present in microgravity.
-Froth formation in agitated solutions makes it difficult to measure their volume.

Additionally, in space, a severe hemorrhage may demand an immediate surgical intervention before diagnostics may have localized the source of bleeding (Kirkpatrick et al., 2001; Doarn 2007; Kirkpatrick et al., 2017a). A group of flight surgeons, trauma surgeons, and biomedical engineers emphasized that laparotomy could be required to stabilize a patient prior to further procedures (Doarn 2007; Ball 2014; Lamb et al., 2014; Kirkpatrick et al., 2017a).

Damage control surgery (DCS) (Doarn 2007; Ball 2014; Lamb et al., 2014) should be used to provide surgical control of hemorrhage. However, DCS should be performed in association with Damage Control Resuscitation (DCR) (Doarn 2007; Ball 2014; Lamb et al., 2014; Kirkpatrick et al., 2017b; Chang et al., 2017). This paradigm states that essential surgery is needed to preserve the physiological reserves of patients implementing only the necessary tasks by means of a few, selected procedures. Besides limiting the procedures to the essential, this method does not require large equipment outlays. A special protocol for austere environment is the Remote Damage Control Resuscitation (RDCR) (Kirkpatrick et al., 2017b; Chang et al., 2017), a treatment strategy for the severely injured trauma patient, designed to limit hemorrhage and produce or preserve adequate levels of physiological reserves for the DCS in the prehospital phase (Kirkpatrick et al., 2017b; Chang et al., 2017). For RDCR on Earth, the doctrine of permissive hypotension has been adopted (Lamb et al., 2014). This practice aims at limiting ongoing hemorrhage by reducing pressure while maintaining a “critical level” of vital organ perfusion. In this approach, few signs are necessary to evaluate the patient status such as the presence of a palpable radial pulse, mental status, and a systolic blood pressure (SBP) of 80–100 mmHg. Traumatic brain injury (TBI) needs a higher SBP to preserve cerebral perfusion pressure and avoid a secondary ischemic injury to the brain (Kirkpatrick et al., 2017b; Chang et al., 2017). However, applying this strategy during spaceflight would be difficult, especially in missions beyond low Earth orbit (LEO) due to the challenging issue of blood transfusion storage.

The early use of blood products, fresh warm whole blood, and other blood components, as well as other devices (hemostatic dressing, extremity tourniquets, junctional tourniquets, abdominal aortic and junctional tourniquets (AAJT), non-absorbable expandable, injectable hemostatic sponge (XSTAT), resuscitative endovascular balloon occlusion of the aorta (REBOA), intra-abdominal self-expanding foam, tranexamic acid administration, and expandable hemostatic sponges (Gordy et al., 2011; Jenkins et al., 2014; Bjerkvig et al., 2016; Rappold and Bochicchio 2016) is representative of DCR and RDCR. These protocols should be particularly useful in space missions where conditions are extreme rather than only austere. In fact, despite the numerous experiments on the feasibility of emergency procedures in microgravity (Campbell 2002; Dawson 2008; Kirkpatrick et al., 2009c; Alexander 2016; Panesar and Ashkan 2018; Robertson et al., 2020), the complex pathophysiology of hemorrhagic shock is mostly still unknown.

Space missions, in any case, may not allow all the procedures we are accustomed to on Earth, for example, the use of a “massive transfusion” protocol with fresh frozen plasma in conjunction with blood and platelets for a severe ongoing bleeding.

ATLS (Ball 2017) protocols have been adapted to the unique pathophysiological mechanisms (Panesar and Ashkan 2018; Nowak et al., 2019) present in space (Kirkpatrick et al., 2009c), but it is still not known how a severe bleed or cardiac failure might affect the hemodynamic state secondary to microgravitational fluid shifts (Kirkpatrick et al., 2009b). Plus, prolonged fluid infusion may have the effect of draining most of the limited supplies of the crew, adversely affecting the clotting profile and/or induce hypothermia (Kirkpatrick et al., 2009b; Panesar and Ashkan 2018). For limb and extremity trauma, tourniquets or hemostatic dressings may be adequate (Rappold and Bochicchio 2016; Panesar and Ashkan 2018). On the contrary, the region of the trunk cannot be treated by external pressure to control hemorrhage and, so far, hemorrhagic shock. Hemodynamic deterioration may prove difficult to address, owing to homeostatic decompensation, the fact that there is no access to facilities and equipment, or due to the lack of trained staff. As a result, DCS has been introduced also in space (Doarn 2007; Ball 2014; Lamb et al., 2014; Kirkpatrick et al., 2017a). In the exploratory phase, hemostasis and/or control of endogenous bacterial contamination must be achieved.

On the ISS, the only resuscitation fluids available are 4 L of normal saline (Nowak et al., 2019), and methods to generate crystalloids in flight are under investigation (Kirkpatrick et al., 2001; McQuillen et al., 2011). On the other hand, response to reduced gravity on other planets such as Mars, or on the Moon is largely unknown, and no appropriate protocols are available to date.

### Missions to Mars and Other Long-Term Mission Peculiarities for Hemorrhagic Shock Treatment

As already stated, blood transfusion in space depends on, among many conditions, limited vehicle dimensions (Summers et al., 2005; Hamilton et al., 2008; Alexander 2016; Nowak et al., 2019). In fact, during a spaceflight, mass, volume, and the necessary power to preserve stored items are known constraints. In addition, blood transfusion in the microgravity environment presents numerous difficulties. For example, although intravenous cannula infusions, phlebotomy and catheterization are possible, in weightlessness once the CMO and the patient are restrained, liquids have a different behavior than on Earth. Blood collection in microgravity should be possible with the use of a vacuum, syringe, or pump. Infusion could be accomplished with a pressure bag or a syringe (Hamilton et al., 2008; Nowak et al., 2019).

It must be remembered that on deep space exploration missions, communication with mission control may be delayed or impossible and the possibility of evacuation will be largely dependent on distance and trajectory from Earth. Therefore, the crew should be more able to function autonomously (Hamilton et al., 2008; Alexander 2016; Nowak et al., 2019) because even on the ISS in low Earth orbit, emergent evacuation could take more than 24 h (Summers et al., 2005; Hamilton et al., 2008; Alexander 2016).

### Topical Hemostatics in Space

The improved understanding of the coagulation process has produced a growing number of hemostatic agents that can be topically applied (Alexander, 2016; Chiara et al., 2018; Huang et al., 2020; Tompeck et al., 2020). In a systematic review in 2018, Chiara et al. (2018) selected four categories: 1) adhesives (liquid fibrin adhesives and fibrin patch), 2) mechanical hemostats, 3) sealants, and 4) hemostatic dressings (mineral and polysaccharides). Each one of them is described according to their employment and scientific foundation.

Despite their different utilization and activation modality, it must be generally underlined that the first concern of the surgeon is the state of the patient’s endogenous coagulation system. A mechanical agent after surgical hemostasis or packing is the right choice in case of normal coagulation. If the patient’s coagulation cascade is not reliable, the hemostat of choice should be an agent that may be effective even when coagulation factors are not, for example, adhesive products (Chiara et al., 2018). In the case of ongoing arterial or high flow bleeding, a patch-supplemented adhesive agent is indicated, as it is directly applicable under pressure on the site of the bleeding. A sealant agent is useful when the bleeding source is an organ like the liver, pancreas, and kidney, or to close a lung wound. Finally, hemostatic dressings should be considered in junctional and non-compressible hemorrhages, for example, in the neck, groin, or axilla (Chiara et al., 2018).

All these hemostats should be available in hospital service because of their different usages and indications. On the ground, tourniquets, conventional bandages, and advanced hemostatic dressings should be available to stop the bleeding in complex situations (Chiara et al., 2018).

New hemostats (nanotechnology) defined as self-assembling peptide nanofibers and chitosan nanofibers have also been used in clinical applications (Corwin et al., 2015; Chaturvedi et al., 2017; Chiara et al., 2018; Estep 2019; Huang et al., 2020; Tompeck et al., 2020).

### Hemoglobin-Based Oxygen Carriers

Artificial red blood cell substitutes (hemoglobin-based oxygen carriers (HBOCs)) are under study to find a way of providing oxygen delivery and volume expansion in such extreme environmental conditions as deep space missions (Kirkpatrick et al., 2009c; Nowak et al., 2019). Originally, their application was studied to prevent transfusion reactions and/or bloodborne disease transmission. Other issues related to HBOCs need to be mentioned. For example, religious motives against transfusions or the need for a rare blood type. In addition, HBOCs may be used as an alternative to blood products in areas where the usual form of blood donation is not available, such as in space missions far from the Earth’s orbit (Moore et al., 2009; Corwin et al., 2015; Weiskopf et al., 2017; Estep 2019; Nowak et al., 2019). Although HBOCs have many advantages as they can be stored at room temperature and require relatively little preparation, they are liquid and have a mass and volume similar to packed red blood cells. Although Moore et al. (2009) published a systematic review showing there is no statistical difference in the mortality rates of shock patients with placebo and HBOC-treated patients, adverse events have been present, and these substitutes are still under review (Nowak et al., 2019).

### Lyophilized Blood Products

As reported by Nowak et al. in 2019, plasma is the only lyophilized blood component in clinical use (Pusateri et al., 2016; Garrigue et al., 2018; Nowak et al., 2019). It has the advantage to be stored in a powder form at ambient temperatures for up to 2 years, and at the moment of transfusion, it can be reconstituted for infusion within a few minutes. In this way, it is advantageous with respect to the limited shelf life of other products and also with respect to the reduced mass and volume of the lyophilized component. In addition, lyophilization may provide storage of autologous blood products with lower risks for infection and transfusion reaction. However, to date, lyophilization of red blood cells and platelets is still experimental.

### Some of the Hemorrhagic Shock Issues With Unknown Effects in Space

*Bacterial translocation*: To date, the theory suggesting that the gut, when suffering from oxygen debt, starts to leak endotoxin and bacteria systemically which then initiates an inflammatory reaction, has not been studied in space (Lord et al., 2014). On Earth, the severity of organ damage that starts in this way depends on bleeding severity and shock duration. The mesenteric lymphatics seem to be the major conduit for the transport of gut-derived bioactive factors into the systemic circulation (Diebel et al., 2012). Organ damage starts depending on bleeding severity and shock duration. Vital organ hypoperfusion usually begins in the gut and progresses to the kidney, liver, and lungs. The emission of large amounts of damage-associated molecular patterns (DAMPs) in polytrauma, systemically circulating, affects the patient’s whole body as initiators of systemic inflammation that is an exaggerated defense response with consequent organ failure (Wutzler et al., 2013; Huber-Lang et al., 2018; Matheson et al., 2018; Relja et al., 2018; Relja and Land 2020).

*The endothelium* while the linkage between oxygen debt and traditional organ failure (renal, hepatic, lung, etc.) has been long recognized; two additional highly dynamic tissues should be considered: the endothelium and the blood. These can be thought of as an integrated organ system, and are strongly related to oxygen delivery in the body (Lord et al., 2014). Microcirculation approximately represents an area of 4,000–7,000 m^2^ with endothelium being a major target for trauma induced hemorrhage and hypoperfusion damages. When endothelial damage is present, hemorrhage and hypoperfusion therapy can produce damages (reperfusion damages) in the epithelium. In this case, the primary goal in trauma care should be the fast mitigation of oxygen debt (Jenkins et al., 2014).

## Discussion and Conclusion

The current ever renewed interest for space missions, in deep space or to another planet, has brought novel conclusions on astronaut health. Although the risk of hemorrhage is relatively low, the next missions to Mars pose a new set of health challenges. Mars is farther than any planet to which humans have traveled before. From or to it, astronauts will not be able to return in case of a health emergency. Also, repairing any injured area of our body would be impossible and even what we consider a “common condition” could turn out to be a devastating event (Nowak et al., 2019). Any traumatic injury or medical condition could possibly lead to life threatening hemorrhage and may be fatal. Although to date there have been no accounts of major hemorrhage on any space missions, protocols similar to those prepared for austere environments on Earth are needed, given that space is considered the most extreme environment ever experienced. Even if the likelihood of blood loss may be reduced through preventive measures, the occurrence of significant blood loss cannot be completely excluded. Even if blood loss from traumatic injury is more likely to occur during the dynamic stages of flight (launch, landing, and docking, and extravehicular activities (EVA)) (Alexander 2016; Nowak et al., 2019), there are additional risks on Lunar, Martian, or other planetary where gravity creates the possibility of fall and crush injuries. Other causes of nontraumatic blood loss, such as gastrointestinal bleeding from ulceration or sequelae from high-dose radiation exposure, may also be possible.

If the blood transfusion process is challenging in austere environments, where access to stored blood, equipment, and personnel may be limited or nonexistent, to date in space such an event can cause the entire mission to be aborted, or the exhaustion of mission supplies. On Earth, protocols used in mass casualties (SALT: Sort. Assess, Life-saving intervention, Treatment or transport) are helpful tools to decide the strategy of cure and treatment in case of multiple injured people. In space, the bulk of risk mitigation for health issues in low Earth orbit (LEO) is placed more on preventive medicine rather than treatment. The medical protocol (International Space Station ISS Astronaut Medical Treatment Algorithms/Protocols ed US NASA, 2015 (U.S. NASA 2015)) for the ISS is designed to “stabilize and transport” an ill or injured crewmember to reach a definitive medical care facility (DMCF) on Earth (Hamilton et al., 2008). Exploration class missions to the moon have a similar plan, and 4–5 days are needed to transport an ill or injured crewmember to DMCF on Earth (Hamilton et al., 2008).

In case of bleeding, in deep space missions or on Mars, the major issue to face is the absence of a storage possibility for blood and blood derivatives. Blood and its derivates as stated before are the main treatment for hemorrhage. Until today, no resource coming from Earth will last enough to reach the red planet, storage on the spacecraft has limitations, and even in case of a prompt resolution of these problems, a severe trauma requiring transfusion could drain the entire resources of the crew, resources that, at this moment, are not possible to reinstate. This is probably the main problem that needs to be tackled for cure, either surgical or non-operative. Concerns are many: Should the CM O be only a surgeon or at least a physician? What kind of aid could he/she need? A surgical robot, humanoid, or other computerized devices? What degree of autonomy should they have? The delay in communication could be a significant problem, or even the lack of them, just to mention some of them. A great deal of work needs to be done to study and prepare protocols for hemorrhage treatment.

While programs need to be based on proven methods and further studies on hemorrhage control are required before they can be applied in a peculiar environment as space research, hemorrhage control innovations in austere and extreme environments will probably provide the best scenarios to prepare a strategy for missions in deep space.
